# Ultra-Low Oxygen and Preconditioning Storage Regulate Ethylene Synthesis to Prevent Corky Disorders in ‘Fuji’ Apple

**DOI:** 10.3389/fpls.2022.910139

**Published:** 2022-05-31

**Authors:** Camila Riaño, Tomás Ribba, Juan I. Marchant, José A. O’Brien, Carolina Contreras, Juan P. Zoffoli

**Affiliations:** ^1^Departamento de Fruticultura y Enología, Facultad de Agronomía e Ingeniería Forestal, Pontificia Universidad Católica de Chile, Santiago, Chile; ^2^Departamento de Genética Molecular y Microbiología, Facultad de Ciencias Biológicas, Pontificia Universidad Católica de Chile, Santiago, Chile; ^3^Instituto de Producción y Sanidad Vegetal, Facultad de Ciencias Agrarias y Alimentarias, Universidad Austral de Chile, Valdivia, Chile

**Keywords:** hypoxia, ACO, ACS, preconditioning, ethanol, acetaldehyde

## Abstract

Corky disorders in apples represent a significant problem for long-term storage where controlled atmosphere (CA) is mainly used. Ultra-low oxygen (ULO) is an alternative to CA, which consists of low partial pressure of O_2_ to maintain a low metabolism in the apple fruit, achieving an effective decrease in the ethylene production and physiological disorders. The aim of this research was to study the effectiveness of a short hypoxia period on the development of cork physiological disorders during the storage of apple. ‘Fuji’ apples were prestored under ULO (0.5 kPa O_2_) for two periods of time (15 and 30 days) and at two temperatures (0 or 5^°^C). Corky physiological disorders increased at 5^°^C prestorage temperature; however, ULO treatments for 15 or 30 days at 0 or 5^°^C achieved a significant reduction in corky disorders near to 1%, compared with control treatments. In addition, a considerable reduction in ethylene production for up to 30 days was observed in ULO-treated fruit at 0 and 5^°^C. ULO for 30 days at 0 and 5^°^C increased the internal production of ethanol and acetaldehyde, causing a lower sensory quality due to the presence of fermentative flavors in fruit stored at 5^°^C. ULO of 15 days of conditioning decreased the relative expression of ethylene biosynthesis genes *MdACS1* and *MdACO1*, resulting in lower ethylene production.

## Introduction

The worldwide apple production, among other species, is currently led by China, which ranked first among apple producer countries with 40,500 million tons, followed by the EU with 12,227 million tons, the United States 4,671 million tons, and Chile with 1,170 million tons (Statista, 2021). Chile ranks as the leading producer in the Southern Hemisphere with the ∼40% of the total production, with Fuji apple among the top three cultivars produced (Gala 39%, Fuji 14%, and Red Delicious 13%) (Simfruit, 2020; Asociación de Exportadores de Chile, 2021).

Several physiological disorders have been described in Fuji apples, where the corky disorders bitter pit (BP), lenticel blotch pit (LBP), and lenticel breakdown (LB) are the most recurrent (Miqueloto et al., 2014; Argenta et al., 2020). It has been shown that the susceptibility to these disorders increases in larger fruit from young, vigorous trees and during prolonged storage (de Freitas et al., 2015). BP appears during pre- and postharvest and has been associated with calcium (Ca) deficiency in fruits. However, recent studies have indicated that BP incidence is not always correlated with total Ca content, suggesting that the causes of BP are more complex (de Freitas et al., 2015; Falchi et al., 2017; Bonomelli et al., 2020). On the contrary, LBP appears during postharvest, as irregular patches around the lenticels, usually near the calyx end of the apple as in BP symptoms, and the severity increases upon prolonged periods of storage. Less calcium concentration is associated with fruit affected with LBD (Dong et al., 2013). LB is a skin disorder that becomes visible during storage as a round pit concentric to the lenticel with wide subepidermal spaces formed by cell lysis, usually at the shaded sides of the apple in more mature fruit (Tessmer et al., 2016). It has been hypothesized that environmental changes (such as low relative humidity and high temperature) during fruit growth promote microcracks with poor cuticle development favoring the injury to underlying cells, leading to desiccation with further cell breakdown (Turketti et al., 2012; Singh et al., 2016). Postharvest calcium applications reduce BT and LBP (Casero et al., 2009) but increase LB (Singh et al., 2021).

Apples are often stored at low temperatures under controlled atmosphere (CA) with reduced O_2_ and enriched CO_2_. Among the CA techniques, ultra-low oxygen (ULO) is characterized by the reduction in the O_2_ partial pressure to the minimum tolerated by the fruit (hypoxia). For instance, the lowest oxygen tolerated by ‘Delicious,’ ‘Law Rome,’ and ‘McIntosh’ apples is 0.7, 0.9, and 1.9 kPa at 0°C, respectively (Gran and Beaudry, 1993). ULO decreases the respiration rate and ethylene production, delaying fruit ripening and reducing oxidative stress associated with many physiological disorders (Thewes et al., 2015; Oliveira et al., 2018). Lowering the O_2_ partial pressure below the oxygen limit will result in anaerobic compensation, where some fermentation occurs. However, positive physiological effects can also be achieved in the fruit. Studies, carried out on ‘Granny Smith’ apples, demonstrated a reduction in oxidative-related disorder (superficial scald), bitter pit, and lenticel blotch pit, using a combination of 30 days delayed storage at 3°C under ULO partial pressure (0.3–0.5 kPa) after 150 days of storage at 0°C (Zoffoli et al., 2018). Similar results using a short anaerobic period before storage at 0°C alleviated bitter pit and superficial scald in ‘Granny Smith’ and ‘Golden Reinders’ apples (Val et al., 2010). The results showed that the initial ULO treatments reduced the ethylene production, conjugated trienols, and respiration rate of the fruit (Pesis et al., 2010; Val et al., 2010; Zoffoli et al., 2018).

The ethylene biosynthesis pathway has two critical steps: the production of 1-aminocyclopropane 1-carboxylic acid (ACC) *via* the conversion of S-adenosylmethionine (SAM) by ACC synthase (ACS) and then the formation of ethylene from ACC by ACC oxidase (ACO) (Ji and Wang, 2021). Moreover, the conversion of ACC to ethylene is oxygen-dependent. This is due to the ACO reaction mechanism, which needs oxygen as an activator (Houben and Van de Poel, 2019). Thus, an increase (high O_2_) or a decrease (hypoxic conditions) in oxygen availability can affect ethylene biosynthesis (Pegoraro et al., 2012). ULO treatments in apples decrease the production of ethylene, and its suppression decreases the expression of *ACS* genes (Asif et al., 2009). In apple, both *ACS* and *ACO* are multigene families. ACC is considered the rate-limiting compound, and ACS plays an important role in the biosynthesis of ethylene. Thus, the *ACS* gene family in apple is composed by at least six genes: *MdACS1, MdACS2, MdACS3A, MdAC3B, MdACS5A*, *MdACS5B, and MdACS6* (Li et al., 2013). Among these, *MdACS6* catalyzes the ethylene production in the preliminary stage of fruit development and decreases when the *MdACS3A* is initiated (Varanasi et al., 2011; Li et al., 2015); only *MdACS1* and *MdACS3A* are specifically expressed in fruit tissue and have important roles in the regulation of fruit ripening. *MdACS1* is highly expressed after fruit ripening and is responsible for the burst of ethylene production in System 2 (Tan et al., 2013). On the contrary, the *ACO* gene family is composed of seven genes: *MdACO1-7* (Houben and Van de Poel, 2019). All ACO enzymes are O_2_-dependent (Wiersma et al., 2007; Li and Yuan, 2008; Li et al., 2013; Yang et al., 2013). Of all *MdACO* genes, *MdACO1* has been highlighted as one of the most relevant for ethylene synthesis in fruits as shown by Schaffer et al. (2007), where transgenic lines downregulating *MdACO1* result in low ethylene levels.

Ethylene has been known for decades to function as a regulator to flood stress responses to avoid or delay hypoxia; however, new findings place ethylene as a mediator in hypoxia acclimation and anticipation (Hartman et al., 2021). Most studies on hypoxia and biosynthesis of ethylene-controlled genes have involved vegetable crops concerning floods or waterlogged plants, finding an ethylene-mediated response to hypoxia. However, ethylene rarely enhances hypoxia-adaptative genes or improves hypoxia survival in plants (Hartman et al., 2020). In apple fruit, hypoxia appears to have no effect on the timing of ripening, but it decreases ethylene production and volatiles (Lumpkin et al., 2014) and negatively affects transcript accumulation of *ACS* and *ACO* genes (Cukrov et al., 2016).

Considering all this information, we hypothesize that an initial short exposure at ULO concentration (conditioning treatments) alleviates the incidence and severity of corky disorders and modulates the ethylene biosynthesis genes *MdACS* and *MdACO* at a transcriptional level in Fuji fruit. This study aimed to evaluate the effect of ULO conditioning treatments (initial ULO for 15 or 30 days at 5°C) on the appearance of physiological disorders (BP and LBP) and on the gene expression behavior of ethylene biosynthesis *MdACS* and *MdACO* in Fuji apples.

## Materials and Methods

### Plant Material, Treatments, and Storage Conditions

**FIGURE 1:**
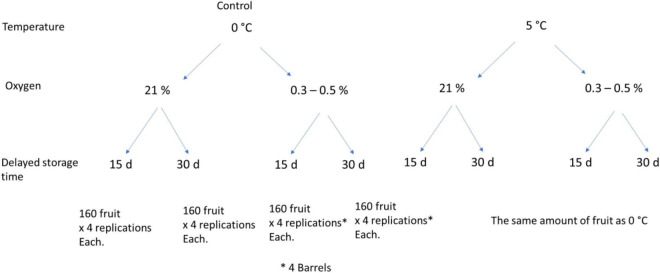
Fruit distribution (5,120 total) in the four conditioning treatments (delayed storage at 5°C at 21 and 0.3–0.5 kPa O_2_ for 15 and 30 days) and control (stored directly at 0°C at 21 and 0.3–0.5 kPa O_2_ for 15 and 30 days).

Fuji apples were handpicked on April 6, 2018, from a commercial orchard located in Longaví in Maule Region, the central valley of Chile (36°00′56.4′′S, 71°40′53.0′′W). The commercial production uses Fuji/MM106 rootstock combination with Granny Smith as pollinizer. The fruit was selected between sizes 70 and 100 healthy and without external defects. The fruit was then transported to the Postharvest Laboratory of Universidad Católica de Chile in Santiago (308 km apart) and stored overnight at 0°C. An initial fruit maturity was assessed on 40 randomly selected fruit. Then, the remainders (5,120 fruit) were grouped randomly into eight sets or treatments of 640 fruit. Four groups were stored at 5°C as conditioning treatments, and the remaining groups were left as control treatment at 0°C. Conditioning treatments included a prestorage treatment with 0.3–0.5 kPa O_2_ for 15 and 30 days at 5°C. These treatments were compared with control fruit stored directly at 0°C considering ULO or control atmosphere (21 kPa O_2_, 0.03% kPa CO_2_). The conditioning treatments were done in independent barrels, with four barrels per treatment or replications, and 160 fruit per barrel. Under regular atmosphere, the untreated control fruit was placed in boxes (four boxes as subsamples, per replication) and stored at 0 and 5°C. Details of treatments and fruit amount are summarized in Figure 1.

After the conditioning treatments were completed, the fruit was packed in cardboard boxes and stored to complete 90 days at 0°C in standard atmosphere (21 kPa O_2_, 0.03% kPa CO_2_).

Ultra-low oxygen conditioning treatments were applied in 200 L-sealed plastic barrels with a sampling port and a septum attached to facilitate gas monitoring. The fruit was stacked in plastic crates, placed inside the barrels (four boxes per barrel with 160 fruit in total), hermetically sealed to avoid gas leakage, and flushed with gaseous nitrogen until reaching an O_2_ pressure below 0.5 kPa. Each barrel represented a replicate. In order to obtain the desired partial oxygen (0.3–0.5 kPa O_2_) and CO_2_ (<0.5 kPa) pressure, initial air (21 kPa) was replaced with 100 kPa N_2_, dispensed from a nitrogen cylinder (Indura S.A., Santiago, Chile). The oxygen partial pressure was adjusted manually following the methodology described by Zoffoli et al. (2018), by flushing with nitrogen (to lower it) or with air (to raise it). Gas monitoring, O_2_, and CO_2_ were performed using a portable MAP Test Gas Analyzer 4000 (Hitech Instruments Ltd., Luton, United Kingdom). After applying all treatments, ULO and control fruit were removed from the barrels, packed in 19-kg boxes per treatment without plastic bags, and stored at 0 ± 0.5°C until complete 90 days, followed by an additional ripening period of 9 days at 20°C.

### Ultra-Low Oxygen Monitoring Inside the Containers

To measure the concentration of O_2_ and CO_2_ in the plastic barrels, a MAP Test Gas Analyzer 4000 was used (Hitech Instruments, Ltd., Luton, United Kingdom) with zirconia and katharometer sensors, respectively. A 20-mL sample of free air space from the container was extracted with a syringe and injected into the MAP Test Gas Analyzer. Two measurements were carried out daily, adjusting the O_2_ concentration to the required levels (0.3–0.5 kPa). CO_2_ partial pressure was kept at <1 kPa using hydrated lime. According to the gas pressure monitoring and manual adjustment performed during the 15 or 30 days of conditioning, the O_2_ concentration was adjusted between 0.3 and 0.5 kPa.

### Fruit Quality Assessments

At harvest, apple fruit had a starch index value of 8, 76.8 N of firmness, 14.5% of total soluble solids (TSS), and 0.25% of titratable acidity (TA). Quality parameters were assessed after 15 and 30 days at 0 and 5°C (conditioning), 30 days at 0°C and 5°C, 90 days of storage at 0°C, and a ripening period of 9 days at 20°C.

Firmness was measured on two opposite sides of the equatorial region of each fruit after peel removal, with a penetrometer (model FT 327; Effegi, Milan, Italy) fitted with an 11.1-mm plunger. TSS was measured by extracting fresh juice from each apple using a press and a PAL-1 thermocompensated digital refractometer (Atago, Japan). TSS values were expressed in%. TA was determined in a sample of 5 mL of juice per replicate, adding 5 mL of distilled water and then titrating with NaOH 0.1 N until reaching a pH of 8.2 using a digital pH meter pH 211 (Hanna instrument, Woonsocket, RI, United States). Values were expressed in% of malic acid. Starch content was rated visually with the iodine test according to the CTIFL (Center Technique Interprofessionnel des Fruits et Légumes, Paris, France) scale from 1 to 10, 1 = non-starch degradation or immature fruit and 10 = total degradation or overmature fruit. A total of five fruits were measured per replication, with four replications per treatment. The ethylene production was determined by placing one apple fruit in a 900-mL flask hermetically closed for 2 h. A sample of 100-μL microsyringe was taken from the flask and injected into a GC8340 gas chromatograph (Fisons Instruments Inc., MA, United States) equipped with a glass column packed Cromosorb 102 (Thermo Electron Corporation, Milan, Italy) to quantify ethylene. Ethylene concentration was calculated using a certified standard gas (5% ethylene) and expressed as μL kg^–1^h^–1^. On the contrary, respiration or CO_2_ production rate was measured by extracting 20 mL of air from the airtight container and immediately injected in a gas meter MAP Test Gas Analyzer 4000 (Hitech Instruments, Ltd., Luton, United Kingdom). CO_2_ production was obtained in percentage and expressed as mL of CO_2_ kg^–1^h^–1^. The ethylene concentration and respiratory rate were determined in three fruit per replicate, and four replicates for treatment.

### Corky Physiological Disorder Assessments

Physiological disorders were evaluated visually after 90 days of storage at 0°C and after 9 days at 20°C, following the description of the symptoms published in Tree Fruit Research and Extension Center of Washington State University (2007). Incidence of bitter pit and lenticel blotch pit was calculated as a percentage of affected fruit from a total fruit. Severity was evaluated as the percentage of area affected in the whole fruit with the symptoms of corky disorders (0–100%).

### Ethanol and Acetaldehyde Determination

The accumulation of ethanol and acetaldehyde in the fruit results from the anaerobic conditions induced by the storage during short periods under low oxygen treatments. The evaluation of alcohol production was one of the main variables evaluated in this study. The alcohol concentration of apples was carried out at 0, 15, and 30 days of conditioning, 90 days of storage at 0°C, and 9 days at room temperature (20°C) in three fruits per replication for each treatment. Each apple was cut into eight slices and weighed, and its juice was extracted. The total volume extracted was measured in a test tube, to be later used in the calculation of the alcohol concentration. 10 mL of juice was placed into a 20-mL vial with 2 g of NaCl. The vials were sealed with a crimper and shaken with a vortex for 30 s until the sample was homogenized. Later, the samples were incubated in a water bath for 40 mins at 60°C following the methodology described by Mitcham and McDonald (1993). Finally, the air sample from the vial was extracted with a 500-μL microsyringe and measured in a gas chromatograph, GC-2014 (Shimadzu, Japan) equipped with a flame ionization detector (FID) at 250°C and RTX capillary column (Restek Corp. Bellefonte, PA, United States). The values identified as the area under the curve at 1.17 min (acetaldehyde) and 1.22 min (ethanol) were then quantified by comparison with peak areas of standard solutions of known concentrations. Values were transformed for both ethanol and acetaldehyde and expressed in μmol mL^–1^.

### Sensory Tests

Two acceptability tests were carried out to assess the ULO treatment effect in the apple flavor perceived by the consumers concerning a fermentative flavor. Panelists were asked to score acceptability using two categories to rank samples between 1 (normal flavor) and 2 (fermentative flavor). The first test was carried out on fruit from the treatments with ULO at 30 days (treatment numbers 5, 6, 7, and 8). The presence of fermentative flavor was not verified at 15 days since only four treatments left the preconditioning at this date, while at 30 days, all the treatments left their preconditioning time, which allowed to determine the effect of a long time of exposure to low oxygen. The second test consisted of an acceptability test performed with fruit from all treatments after 90 days of storage at 0°C (treatment numbers 1–8). After removal from storage, the fruit was held at 20°C for 24 h before the sensory assessment. A panel composed of 35 sensory assessors was used for the first test and 31 for the second one. Students, staff, and academics were recruited from the Department of Horticulture and Forestry Engineer of the Universidad Católica of Chile. In each test, the panelists were presented with six pairs of samples, showing 12 samples total per panelist, according to Meilgaard et al. (2007). One pair at a time was presented. Each sample was previously identified with a random three-digit code, and each panelist was asked to identify a “strange” flavor per pair. Each sample consisted of an 8-g apple slice with the peel attached. In the second test, due to the number of treatments (1–8), not all of them were presented to each panelist, but rather these were randomly split, distributed, and presented to different panelists the same number of times. In other words, all treatments were assessed an equal number of times, but panelists did not taste all treatments. This procedure was done to avoid the excessive number of samples for each panelist. All treatments were evaluated within the same day.

### Total RNA Extraction and Real-Time Quantitative PCR

Analysis of the expression level of ethylene biosynthesis genes was carried out in ‘Fuji’ apple fruit at 0, 15, and 30 days of ULO conditioning treatments, 90 days of storage at 0°C, and 9 days of ripening at room temperature in three fruits per replicate for each treatment. This analysis was done on two treatments with the most contrasting results in ethylene production (control for 15 days at 5°C and ULO for 15 days at 5°C). Flesh fruit samples without peel were frozen in liquid nitrogen and stored at −80°C for further RNA extraction process. The RNA extraction procedure was the Hot borate method and performed according to Wan and Wilkins (1994) with some modifications which included the amount of frozen tissue used (3 g), the amount of PVP (4 g), first incubation at 42°C for 90 mins, and first centrifugation at 12.000 g for 25 min at 4°C. The samples were stored overnight at 4°C to allow RNA precipitation. RNA was recovered by centrifugation at 18,000 g for 45 min at 4°C. The supernatant was discarded, the pellet washed twice with 80% ethanol, and centrifuged at 13.000 rpm at 4°C. After each extraction, the RNA was quantified using a Qubit 3.0 following the manufacturer’s recommendations. Then, a 1% agarose gel electrophoresis was performed to corroborate the integrity of the RNA. All RNA extracts were then treated with DNase I using a reaction protocol of 10X solution (New England Lab, Ipswich, MA, United States). The mixture was incubated at 37°C for 10 min. To inactivate the enzyme, 1 μl of EDTA (5 mM) was added and finally incubated at 75°C for 10 min. cDNA synthesis was performed using the M-MuLV reverse transcriptase (RT) enzyme (New England Lab) coupled to DNase treatment according to the manufacturer’s instructions, using a mix (2:1) of random primers and Oligo-dT.

Quantitative PCR (qPCR) was performed on a StepOnePlus™ real-time PCR system (Applied Biosystems, Bedford, MA, United States) using Hot FIREPol^®^ kit EvaGreen^®^ qPCR (Solis BioDyne) following the manufacturer’s instructions. The reaction was performed in 0.1 mL tubes MicroAmp^®^ Fast 8-Tube Strip. The program used for the amplification was as follows: 95°C for 12 mins, followed by 45 cycles at 95°C for 15 s, 58°C for 20 s, and 72°C for 20 s. A melting curve was constructed at the end of all the runs to assess the specificity of the primers with 0.5°C increases. Primers designed for this experiment are shown in Supplementary Table 1. Relative expression was determined using LinRegPCR software, considering the calculation of primer and reaction efficiency (Ruijter et al., 2009) using the previously reported housekeeping gene *MdGAPDH* (Andre et al., 2016).

### Statistical Analysis

A 3 × 2 factorial experimental design was applied, with three factors and two levels for the O_2_ concentration (Regular and ULO), temperature (0 and 5°C), and period of conditioning (15 and 30 days). The statistical analyses of fruit quality parameters, physiological disorders incidence and severity, and acceptability test data were carried out using SigmaPlot version 14.0 2018 (SYSTAT Software Inc., San Jose, CA, United States). A Tukey test separated differences among treatment means with a *P*-value ≤ 0.05. For the molecular analyses, an ANOVA was carried out with GraphPad Prism 8.0 and multiple comparison of means using the Tukey test (*P*-value ≤ 0.05).

## Results

### Effect of Ultra-Low Oxygen Conditioning on Fruit Quality

The individual effects of the conditioning factors (oxygen, temperature, and period of time) and their interaction on fruit quality parameters after 90 days at 0°C are shown in Table 1. Significant interaction was found between conditioning under ULO and temperature (0 or 5°C) on fruit firmness. In addition, the temperature of the conditioning treatments had a significant effect on the accumulation of TSS and starch degradation.

**TABLE 1:** Individual and interaction effects of ultra-low oxygen (ULO) conditioning treatments: oxygen concentration (0.5 and 21 kPa), temperature (0 and 5°C), and time (15 and 30 days) on Fuji apple quality parameters after 90 days at 0°C.

	Firmness		TSS		TA		Starch degradation	
**Source of variation**	***F* value**	***P* > *F***	***F* value**	***P* > *F***	***F* value**	***P* > *F***	***F* value**	***P* > *F***

Oxygen	1.639	0.2128	1.588	0.2197	0.282	0.60005	0.054	0.81812
Temperature	7.172	0.0132*	4.245	0.0504*	3.459	0.07522	10.595	0.00336**
Time	5.256	0.0309*	0.048	0.8277	2.541	0.12400	0.486	0.49220
Oxygen × temperature	5.741	0.0247*	3.611	0.0695	2.541	0.12400	0.054	0.81812
Oxygen × time	0.355	0.5571	3.461	0.0751	0.071	0.79275	0.216	0.64613
Temperature × time	0.929	0.3448	0.116	0.7368	0.282	0.60005	0.054	0.81812
Oxygen × temperature × time	0.907	0.3505	0.548	0.4664	8.541	0.00745**	0.000	100.000

*P < 0.05, **P < 0.01.

The three factors had a synergic effect on acidity (Supplementary Table 2). Conditioning the fruit at 5°C reduced fruit firmness to 65.3 N compared with control fruit without conditioning that achieved a value of 70.2 N, similar to the firmness of fruit conditioned under ULO at 0°C (Supplementary Table 2A). For TSS, a higher conditioning temperature (5°C) significantly decreased the sugar content in the fruit compared to the fruit conditioned at 0°C (Supplementary Table 2B).

After 9 days of ripening at 20°C, the individual effects of conditioning factors and their interaction on fruit quality parameters are shown in Supplementary Tables 3, 4. In the case of firmness, there was a significant interaction of temperature and time of conditioning (Supplementary Table 4A), while for the TSS was found a synergic effect of the three factors, evidencing that fruit conditioned at 5°C had a lower accumulation of sugars (Supplementary Table 4B). Regarding TA, the ULO conditioning treatments had a significant effect, showing that fruit under lower oxygen had a higher acidity level than the control treatments (Supplementary Table 4C). In terms of starch content, there was a total degradation in all treatments showing the over ripen stage of the fruit.

### Effect of Ultra-Low Oxygen Conditioning on Ethylene Production and Respiration Rate

**FIGURE 2:**
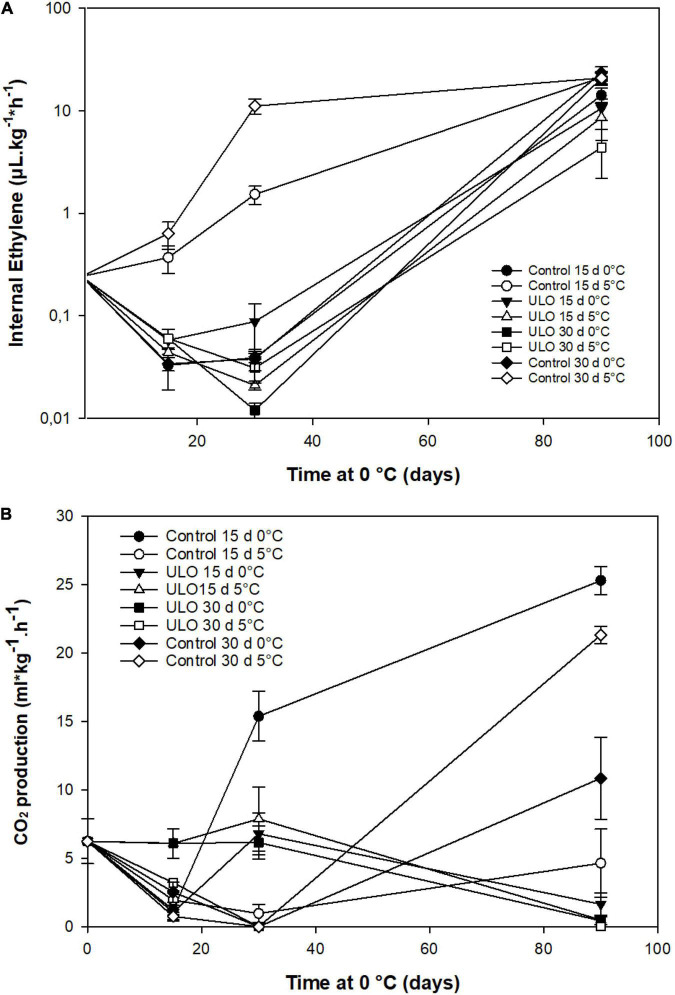
Effect of ultra-low oxygen (ULO) conditioning treatments for 15 and 30 days at 0 or 5°C on internal ethylene production **(A)** and respiratory rate **(B)** of ‘Fuji’ apples stored for 90 days at 0°C, plus 9 days of ripening at 20°C. Values are means ± standard error, n = 4 replicates. ULO, ultra-low oxygen conditioning treatment; Control, conditioning temperature treatment under air atmosphere.

The effect of conditioning treatments on respiration rate and ethylene production was evaluated during storage. Regarding the internal production of ethylene, control fruit that was kept at 5°C for 15 or 30 days showed the highest ethylene production rate compared to the other treatments showing range values of 1 and 10 μL kg^–1^h^–1^. In contrast, ULO-treated fruit at 5°C showed values lower than 0.1 μL kg^–1^h^–1^ even below the harvest reference values (0.25 μL kg^–1^h^–1^). The same pattern was observed in fruit at 0°C (Figure 2A). However, the production of ethylene increased in all treatments after the fruit was removed from ULO, reaching almost the same values among the treatments, and being lower in fruit that was pretreated with ULO at 5°C for 15 or 30 days after 90 days at 0°C (Figure 2A). The respiration rate varied with conditioning treatments at low oxygen and temperature. As shown in Figure 2B, at 30 days of conditioning, the CO_2_ production showed two patterns with significantly higher values in all ULO treatments (5–8 mL kg^–1^h^–1^), while in most of control treatments (except 15 days at 5°C), CO_2_ production was near zero (<1 mL kg^–1^h^–1^). At 90 days, the respiratory rate pattern showed an opposite pattern, the fruit to which ULO was applied presented a strong inhibition of CO_2_ production <0.5 mL kg^–1^h^–1^, whereas control fruit produced higher amounts of CO_2_, especially those that were conditioned for 15 and 30 days at 5°C with values of 25 and 21 mL kg^–1^h^–1^, respectively.

After fruit storage removal and 9 days at 20°C, the ethylene production increased significantly with no differences among treatments. The CO_2_ production decreased in most treatments except for 15 days at 0°C and ULO for 15 days at 0°C (Supplementary Table 5).

### Effect of Ultra-Low Oxygen Conditioning on Physiological Disorders

**FIGURE 3:**
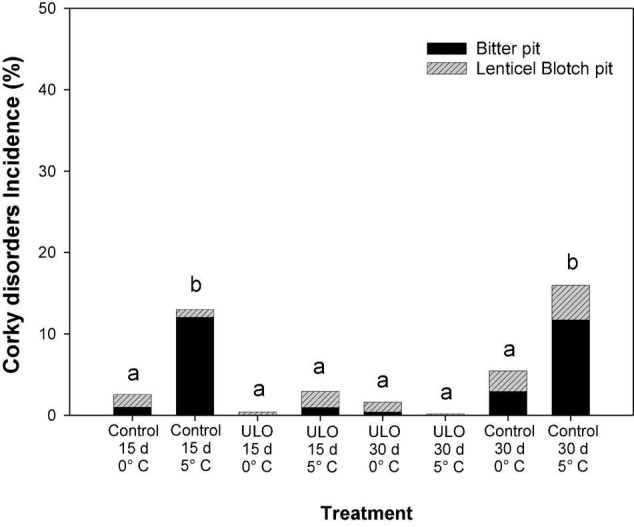
Effect of ULO condition treatments for 15 and 30 days at 0 or 5°C on incidence of bitter pit and lenticel blotch pit after 9 days of ripening at 20°C in ‘Fuji’ apples previously stored for 90 days at 0°C. Values are means, n:4 replicates. Means with different letters are significantly different based on Tukey’s test (*P* ≤ 0.05). ULO, ultra-low oxygen conditioning treatment; Control, conditioning temperature treatment under air atmosphere.

The effect of conditioning on incidence of physiological disorders such as BP and LBP is summarized in Table 2 and Figure 3. For BP, at 90 days of storage, the interaction between ULO and temperature was significant showing a synergistic effect on control of BP (*P-value* < 0.0001). However, the interaction of oxygen and time was not significant (*P-value* = 0.498). Finally, the interaction of the three factors does not affect the control of BP (*P-value* = 0.518; Table 2). For LBP, the interaction of temperature (0 or 5°C) and time of conditioning (15 or 30 days) under ULO affected the incidence of LBP (*P-value* = 0.00369). Hence, the conditioning at 5°C for 30 days induced the highest susceptibility; however, when the period of conditioning was under ULO, the incidence was reduced to the lowest value (data not shown).

**TABLE 2:** Individual effect and interactions of conditioning treatments: oxygen concentration (0.5 and 21 kPa), temperature (0 and 5°C), and conditioning time (15 and 30 days) in ‘Fuji’ apples for the incidence of bitter pit (BP) stored during 90 days at 0°C.

	BP 90 days at 0°C		LBP 90 days at 0°C	
Source of variation	*F* value	*P* > *F*	*F* value	*P* > *F*
Oxygen	75.624	<0.0001***	9.479	0.00514**
Temperature	44.216	<0.0001***	1.667	0.20901
Time	0.705	0.0410	3.129	0.08960
Oxygen × temperature	46.262	<0.0001***	0.074	0.78798
Oxygen × time	0.472	0.498	11.366	0.00253**
Temperature × time	1.167	0.291	0.000	0.98370
Oxygen × temperature × time	0.430	0.518	10.345	0.00369**

*P < 0.05, **P < 0.01, ***P < 0.001.

The total incidence of corky disorders (BP and LBP) evaluated after 90 days a 0°C plus 9 days at 20°C in all treatments is shown in Figure 3. Regarding the conditioning treatments, the ULO treatments for 15 or 30 days significantly reduced the corky disorders to <1% incidence. On the contrary, the control treatments at 5°C for 15 or 30 days presented the highest incidence (13–15%), similar to what was observed at 90 days at 0°C. After 90 days of storage, the severity of the corky disorders showed no significant differences between treatments (data not shown).

### Sensory Test, Ethanol, and Acetaldehyde Concentration

**FIGURE 4:**
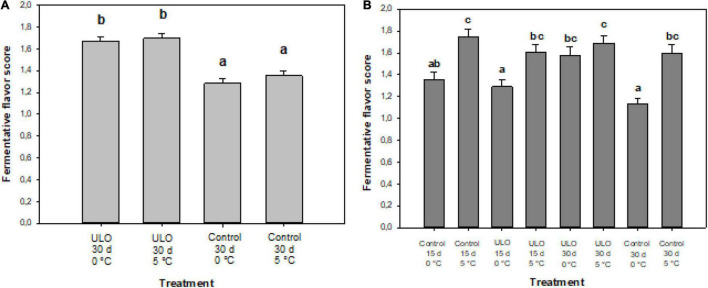
Fermentation flavor score in ‘Fuji’ apple affected by conditioning under ULO for 15 or 30 days at 0 or 5°C and evaluated after **(A)** 30 days of preconditioning and **(B)** 90 days at 0°C. Scores were taken as 1 (normal flavor) and 2 (fermentative flavor). Means with different letters are significantly different according to Tukey’s multiple comparison test (*P* ≤ 0.05).

The conditioning temperature was significant for the development of a fermentative flavor. The interactions of partial pressure of O_2_ and temperature, and partial pressure of O_2_ and time of conditioning were significant (Table 3), indicating that the conditioning under low oxygen with a higher temperature or longer conditioning time triggers fermentation, and therefore, developing the fermentative flavor. The fermentative flavor evaluated in ‘Fuji’ apple fruit after the conditioning is shown in Figure 3. The panelists identified a fermentative flavor produced by the anaerobic process. The 30-day period at ULO preconditioning showed a greater perception of fermentative flavor (1.7 value) versus those conditioned at air atmosphere (1.3 value) (Figure 4A). At 90 days of storage, the temperature showed the most consistent effect; in this case, the conditioning at 5°C showed fermentative scores higher than the control treatments suggesting that consumers perceived a significantly higher fermentative flavor in the samples stored at 5°C (Figure 4B). After 90 days at 0°C, the lowest score for fermentation was found on fruit that was stored directly to 0°C; in the other cases where conditioning treatments were carried out under air or ULO, the sensory evaluation showed a fermentation flavor that increased the value to 1.6–1.8 (Figure 4B).

**TABLE 3:** Individual effect and interactions of conditioning treatments: oxygen concentration (0.5 and 21 kPa), temperature (0 and 5°C), and conditioning time (15 and 30 days) in ‘Fuji’ apples for fermentative flavor stored during 90 days at 0°C.

	Fermentative flavor 90 days at 0°C	
**Source of variation**	***F* value**	***P* > *F***

Oxygen	2.526	0.113
Temperature	40.417	<0.001**
Time	0.000	1,000
Oxygen × temperature	6.238	0.013*
Oxygen × time	14.899	<0.001**
Temperature × time	0.206	0.650
Oxygen × temperature × time	1.289	0.257

*P < 0.05, **P < 0.01.

**FIGURE 5:**
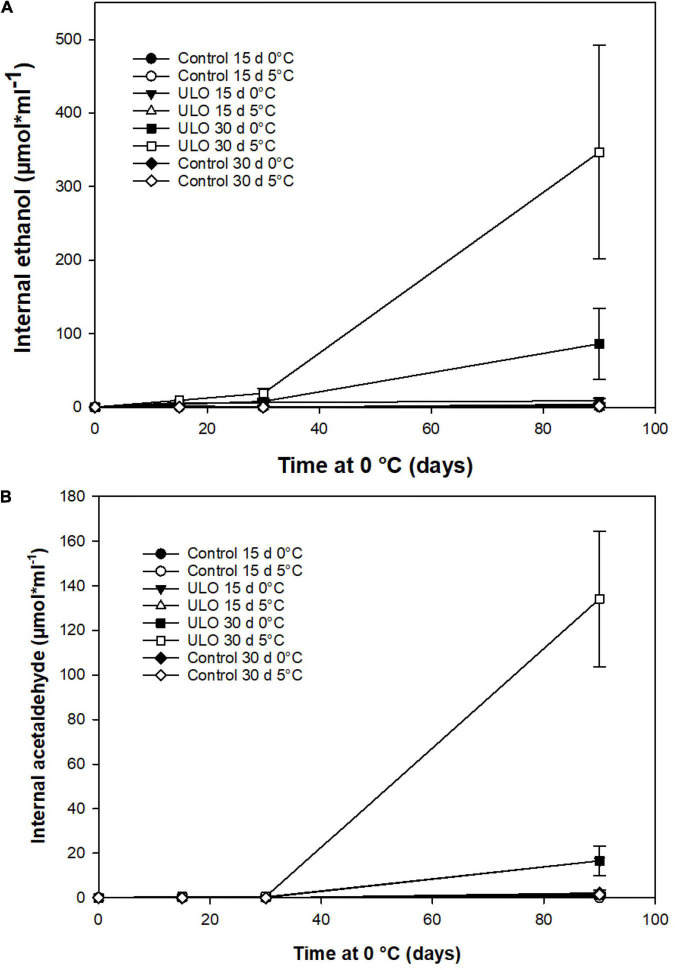
Effect of ULO conditioning treatments for 15 and 30 days at 0 or 5°C on internal concentrations of ethanol **(A)** and acetaldehyde **(B)** in ‘Fuji’ apples stored for 90 days at 0°C. Values are means ± standard error, n = 4 replicates. ULO, ultra-low oxygen conditioning treatment; Control, conditioning temperature treatment under air atmosphere.

Under fermentative metabolism, ethanol and acetaldehyde synthesis had a marked increase. A short conditioning period under ULO produced an accumulation of ethanol and acetaldehyde that extended up to 90 days at 0°C (Figure 5). From the harvest time to 90 days of storage, the internal concentration of ethanol increased progressively in ULO-treated fruit for 30 days either at 0 or 5°C, reaching 3 months of storage values of 90 and 350 μmol mL^–1^, respectively. The ULO treatment for 15 days did not exceed 12 μmol mL^–1^ of ethanol. Control treatments showed the lowest levels of ethanol near 0 μmol mL^–1^ (Figure 5A). After 9 days of ripening at 20°C, there was a sharp decrease in ethanol concentration, reaching similar levels shown by the rest of the treatments (data not shown). On the contrary, acetaldehyde concentration followed the same behavior pattern observed in ethanol levels when the fruit was stored for 30 days under ULO treatments at 0 or 5°C (Figure 5B). The ULO treatment for 30 days at 5°C presented the highest concentration of ∼135 μmol mL^–1^, followed by ULO for 30 days at 0°C with 16 μmol mL^–1^ after 90 days of storage at 0°C (Figure 5B).

### Effect of Ultra-Low Oxygen Conditioning on the Expression of Ethylene Biosynthesis Genes MdACS and MdACO

**FIGURE 6:**
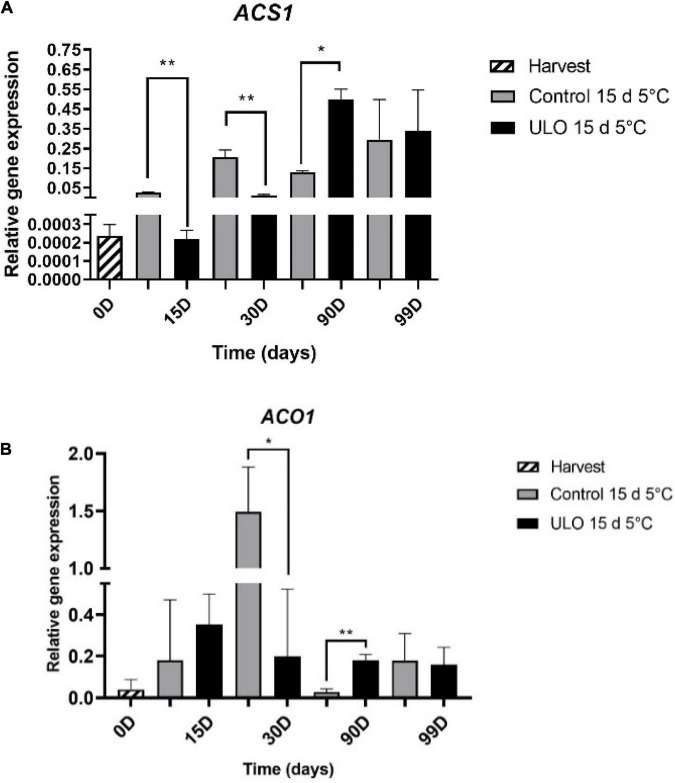
Relative expression of genes involved in ethylene synthesis pathway: *MdACS1* **(A)** and *MdACO1* **(B)**. Data were obtained in cortical tissues of ‘Fuji’ apple fruit from ULO and control at harvest, 15 and 30 days of conditioning at 5°C, 90 days of storage at 0°C, and 9 days of ripening at 20°C. Expression level was measured relative to that of *MdGAPDH*. Bars indicate standard error significantly different according to Tukey’s test *P < 0.05, **P < 0.01.

Considering the critical role of *MdACS1* and *MdACO1* in the autocatalytic burst of ethylene production during fruit development and ripening (Li and Yuan, 2008), the relative expression of *MdACS1* and *MdACO1* genes was evaluated. We selected the most contrasting treatments according to ethylene production (control for 15 days at 5°C and ULO for 15 days at 5°C). As shown in Figure 6, under control conditions, both *MdACS1* and *MdACO1* are upregulated after 15 and 30 days of storage. Interestingly, the upregulation of both genes under ULO at 15 and 30 days of storage is significantly lower when compared to control. At 90 days of storage, the opposite trend was observed, where both genes were upregulated in ULO compared to control.

## Discussion

Bitter pit and LBP are physiological disorders that affect the postharvest quality of apples. These disorders are generally attributed to calcium deficiency and commercially remedied through several calcium applications during fruit development (Raese and Drake, 2002; Val et al., 2008; Casero et al., 2009) or under postharvest applications as soon as the fruit is harvested (Conway et al., 2002). Postharvest treatments vary in effectiveness in reducing these disorders. Prestorage treatment at 10°C, for different times, increased the incidence of bitter pit (Watkins et al., 2004). Similarly, 1-MCP did not control the bitter pit in ‘Golden Delicious’ apples (Gago et al., 2015) and enhanced LBP disorder in ‘Granny Smith’ (Calvo and Cardan, 2010).

In this research, the incidence of corky disorders (BP + LBP) doubled in fruit prestored for 15 or 30 days at 5°C compared to the direct storage at 0°C and evaluated at ripening after 90 days at 0°C. However, the incidence was almost entirely alleviated in ULO-treated fruit (0.3–0.5 kPa O_2_) during the delayed period of storage. These results confirm previous reports (Pesis et al., 2010; Val et al., 2010; Zoffoli et al., 2018) where combination of low O_2_ with high temperature or high temperature alone alleviates these disorders. Although the ULO conditioning treatment was applied for a short period of time, the effectiveness extended 75 days at 0°C after the ULO treatment. The beneficial effect of the ULO prestorage period has been demonstrated and commercially used to reduce oxidative-related disorders. For instance, superficial scald is associated with reactive oxygen species linked to increased gene expression of reactive oxygen species (ROS) scavenging enzymes (Sabban-Amin et al., 2011). ULO involves an initial low oxygen stress render in some level of fermentation including several volatiles, such as ethanol, which has proven to be beneficial for scald control. Acetaldehyde, on the contrary, has proven to produce cross-linkages between proteins, and it is a metabolite more toxic than ethanol; therefore, acetaldehyde seems to be the cause of tissue injury (Pfister-Sieber and Brändle, 1994). As long as the fermentation is not detrimental to normal cellular function, further research is necessary to demonstrate that some metabolites derived from fermentation are involved in ameliorating corky disorders.

Hansen et al. (1992) and Tian et al. (2020) reported that the human odor thresholds for ethanol and acetaldehyde were 2.17 and 0.0025 μmol mL^–1^, respectively. In our study, ethanol (0.022 μmol mL^–1^) and acetaldehyde (0.001 μmol mL^–1^) concentrations in control treatment fruit had levels below the odor threshold perception until 30 days of storage (Figures 5A,B, respectively). Then, at 90 days of storage, in the case of ethanol, all ULO treatments and control treatments exposed to 5°C for 15 days were above the levels of the human odor thresholds. As for acetaldehyde, all treatments except control treatment exposed to 5°C for 15 days exceeded the human odor thresholds. Several studies that relate sensory quality in apples and low oxygen storage show high concentrations of ethanol and acetaldehyde. They have been correlated with the appearance of off-flavors in specific apple cultivars (Thewes et al., 2017).

The rapid increase in ethylene production in control conditions after 15 and 30 days of storage directly correlates with the upregulation of *MdACS1* and *MdACO1* (particularly at the 30-day peak of expression) (Figure 5). Interestingly, the low ethylene levels at the same storage days observed under ULO also correlate with lower expression levels of *MdACS1* and *MdACO1*, particularly at 30 days of storage. Moreover, at 90 days of storage, the ethylene levels under control conditions remain stable while under ULO increase significantly to reach the same level of control conditions. The stable levels of ethylene correlate with the stabilization of *MdACS1* expression and the downregulation of *MdACO1* at 90 days. However, under ULO, the expression of *MdACS1* and *MdACO1* is significantly higher when compared to control. This is particularly evident for *MdACS1*, which had an expression peak at 90 days of storage. The relative expression behavior of *MdACS1* and *MdACO1* obtained in this study can be confirmed by previous reports by Sabban-Amin et al. (2011), who found that the application of ULO for 24 weeks in apple Granny Smith reduced the expression of *MdACS* and *MdACO* when compared to control. Altogether, these results suggest that this can be one of the control points that reduce the internal production of ethylene, delaying the maturation and senescence of fruit by decreasing the expression of genes associated with the synthesis of ethylene and its action in apple fruits (Sabban-Amin et al., 2011; Wright et al., 2015; Saquet and Streif, 2017).

In conclusion, the evaluation of physiological parameters, the incidence of corky disorders acceptability and relative gene expression, suggests that a delayed storage of 15 or 30 days at 5°C promotes the development of corky spots during storage. However, this negative effect is suppressed by a period of 15 days under ULO, which inhibits the biosynthesis of ethylene and its levels in fruit, which results in the accumulation of ethanol. It was demonstrated that there is no detrimental effect on acceptability perceived by the consumer regarding the fruit exposed to atmospheric conditions.

## Data Availability Statement

The original contributions presented in the study are included in the article/Supplementary Material, further inquiries can be directed to the corresponding authors.

## Author Contributions

CR did most of the data retrieval and statistical analyses, wrote the first draft after which CC edited and improved it for publication. JZ was the intellectual author of the work. CC and JO’B were the intellectual contributors for the molecular section of the work. TR conducted molecular work and data analysis. JM did ethanol, acetaldehyde, ethylene, and respiration rate analyses. All authors contributed to the article and approved the submitted version.

## Conflict of Interest

The authors declare that the research was conducted in the absence of any commercial or financial relationships that could be construed as a potential conflict of interest.

## Publisher’s Note

All claims expressed in this article are solely those of the authors and do not necessarily represent those of their affiliated organizations, or those of the publisher, the editors and the reviewers. Any product that may be evaluated in this article, or claim that may be made by its manufacturer, is not guaranteed or endorsed by the publisher.
