# Supplementary material for: Ultra-Low Oxygen and Preconditioning Storage Regulate Ethylene Synthesis to Prevent Corky Disorders in ‘Fuji’ Apple
Source: Front Plant Sci. 2022 May 31;13:910139. doi: 10.3389/fpls.2022.910139 (PMC9194684; doi:10.3389/fpls.2022.910139)
Supplement: Supplementary file 1 [file Data_Sheet_1.docx]

Supplementary Material

**Supplementary Table 1.** List of primers used for Real time qPCR.

| **Gene** | **Primer 5’ F** | **Primer 3’ R** | **Amplified size of cDNA (bp)** | **Accession number** |
| --- | --- | --- | --- | --- |
| *MdACS1* | ACTGAAACCGCTCTGGAAGA | CGTTTCTGGTCATTGTGGTG | 115 | L31347 |
| *MdACO1* | GACGATGTCCAGTCCGAAAT | TCGGAAATGTTTGAGGAAGG | 83 | DQ137850 |
| *MdGAPDH* | CTGCCCCCAGCAAGGAT | TGGCTTGTATTCCTTCTCGTTCA | 62 | MDP0000757565 |

**Supplementary Table 2.** Effect of conditioning (ULO or control) and temperature (0 or 5 °C) interaction on: firmness (N) (A); and temperature effect of total soluble solids concentration (%, TSS) (B) on ‘Fuji’ apple after 90 days a 0 °C.

1. **Firmness**

| **Temperature (°C)** | **Conditioning** | |
| --- | --- | --- |
|  | **ULO** | **Control** |
| **0** | 69.1 aB^z^ | 70.2 aB |
| **5** | 68,8 aA | 65.3 aA |

^z^ The numbers in the row and column with different letters had statistical differences. Lower case letters show differences between oxygen partial pressure (rows), and capital letters between temperature of conditioning (columns).

1. **TSS**

| **Temperature (°C)** | **TSS (%)** |
| --- | --- |
| 0 | 13,5* |
| 5 | 12,9 |

* *P*= 0.05

**Supplementary Table 3.** Individual and interactions effects of ULO conditioning treatments: oxygen concentration (0.5 kPa and 21 kPa), temperature (0 °C and 5 °C) and time (15 and 30 days) on quality parameters of ‘Fuji’ apples after 90 days at 0 °C plus 9 days of ripening at 20 °C.

|  | **Firmness** | | **TSS** | | **TA** | | **Starch degradation** | |
| --- | --- | --- | --- | --- | --- | --- | --- | --- |
| Source of variation | F value | *P* > F | F value | *P* > F | F value | *P* > F | F value | *P* > F |
| Oxygen | 27.921 | <0.0001*** | 2.670 | 0.11532 | 9.715 | 0.00469 ** | 1 | 0.327 |
| Temperature | 22.621 | <0.0001*** | 37.717 | 0<.0001 *** | 1.259 | 0.27293 | 1 | 0.327 |
| Time | 21.589 | 0.000102 *** | 3.251 | 0.08396. | 1.881 | 0.18293 | 1 | 0.327 |
| Oxygen x temperature | 5.145 | 0.032588 * | 1.537 | 0.22712 | 1.881 | 0.18293 | 1 | 0.327 |
| Oxygen x time | 3.327 | 0.080617 | 13.413 | 0.00123 ** | 3.497 | 0.07371 | 1 | 0.327 |
| Temperature x time | 11.972 | 0.002034 ** | 6.429 | 0.01816 * | 0.016 | 0.90182 | 1 | 0.327 |
| Oxygen x temperature x time | 1.680 | 0.207236 | 5.600 | 0.02637 * | 0.762 | 0.39146 | 1 | 0.327 |

**Supplementary Table 4.** Effect of conditioning temperature (0 or 5 °C) and time (15 or 30 days) interaction on: firmness (N) (A), triple interaction of conditioning and temperature on total soluble solids concentration (TSS) (B); and conditioning effect on titratable acidity (C) in ‘Fuji’ apples after 90 days at 0 °C plus 9 days of ripening at 20 °C.

1. **Firmness**

| **Time (days)** | **Temperature (°C)** | |
| --- | --- | --- |
|  | **0** | **5** |
| 15 | 71,9 bB^z^ | 67,9 aA |
| 30 | 68,0 bA | 66,6 aA |

^Z^ The numbers in the row and column with different letters had statistical difference. Lower case letters show differences between oxygen partial pressure (rows) and capital letters between temperature of conditioning (columns).

1. **TSS**

| **Temperature (°C)** | **Conditioning** | | | |
| --- | --- | --- | --- | --- |
|  | **ULO** | | **Control** | |
|  | 15 | 30 | 15 | 30 |
| 0 | 14,8 bc^z^ | 12,5 a | 14,1 b | 14,5 bc |
| 5 | 12,6 a | 12,5 a | 12,4 a | 12,9 a |

^Z^ Means in each column followed by the same letters do not differ significantly.

1. **TA**

| **Conditioning** | **TA (%)** |
| --- | --- |
| ULO | 0,16* |
| Control | 0,13 |

* *P*= 0.05

**Supplementary Table 5.** Respiration rate and ethylene production after 9 days of ripening at 20 °C with different ULO and temperature conditioning treatments.

| **Treatment** | **9 days of ripening at 20 °C** | |
| --- | --- | --- |
|  | **Ethylene production (μL ethylene/kg*h)** | **CO_2_ production (mL CO_2_/kg*h)** |
| Control 15 d 0 °C | 19.1 ns | 14.37 b |
| Control 15 d 5 °C | 34.5 ns | 0.78 a |
| ULO 15 d 0 °C | 9.15 ns | 4.89 b |
| ULO 15 d 5 °C | 20.15 ns | 0.00 a |
| ULO 30 d 0 °C | 14.65 ns | 0.00 a |
| ULO 30 d 5 °C | 22.47 ns | 0.00 a |
| Control 30 d 0 °C | 10.92 ns | 0.00 a |
| Control 30 d 5 °C | 26.33 ns | 0.00 a |
